# Metabolic Flow of C6 Volatile Compounds From LOX-HPL Pathway Based on Airflow During the Post-harvest Process of Oolong Tea

**DOI:** 10.3389/fpls.2021.738445

**Published:** 2021-10-22

**Authors:** Zi-wei Zhou, Qing-yang Wu, Zi-xin Ni, Qing-cai Hu, Yun Yang, Yu-cheng Zheng, Wan-jun Bi, Hui-li Deng, Zhen-zhang Liu, Nai-xin Ye, Zhong-xiong Lai, Yun Sun

**Affiliations:** ^1^College of Life Science, Ningde Normal University, Ningde, China; ^2^Key Laboratory of Tea Science in Fujian Province, College of Horticulture, Fujian Agriculture and Forestry University, Fuzhou, China; ^3^Institute of Horticultural Biotechnology, Fujian Agriculture and Forestry University, Fuzhou, China

**Keywords:** oolong tea, C6 compounds, ADH, turn over, hypoxia

## Abstract

Aroma is an essential quality indicator of oolong tea, a tea derived from the *Camellia sinensis* L. plant. Carboxylic 6 (C6) acids and their derivative esters are important components of fatty acid (FA)-derived volatiles in oolong tea. However, the formation and regulation mechanism of C6 acid during postharvest processing of oolong tea remains unclear. To gain better insight into the molecular and biochemical mechanisms of C6 compounds in oolong tea, a combined analysis of alcohol dehydrogenase (ADH) activity, *CsADH2* key gene expression, and the FA-derived metabolome during postharvest processing of oolong tea was performed for the first time, complemented by *CsHIP* (hypoxia-induced protein conserved region) gene expression analysis. Volatile fatty acid derivative (VFAD)-targeted metabolomics analysis using headspace solid-phase microextraction–gas chromatography time-of-flight mass spectrometry (HS-SPEM-GC-TOF-MS) showed that the (*Z*)-3-hexen-1-ol content increased after each turnover, while the hexanoic acid content showed the opposite trend. The results further showed that both the ADH activity and *CsADH* gene expression level in oxygen-deficit-turnover tea leaves (ODT) were higher than those of oxygen-turnover tea leaves (OT). The C6-alcohol-derived ester content of OT was significantly higher than that of ODT, while C6-acid-derived ester content showed the opposite trend. Furthermore, the HIP gene family was screened and analyzed, showing that ODT treatment significantly promoted the upregulation of *CsHIG4* and *CsHIG6* gene expression. These results showed that the formation mechanism of oolong tea aroma quality is mediated by airflow in the lipoxygenase–hydroperoxide lyase (LOX-HPL) pathway, which provided a theoretical reference for future quality control in the postharvest processing of oolong tea.

## Introduction

Oolong tea, which is derived from the *Camellia sinensis* L. plant, is a well-known traditional Chinese tea. It is favored by consumers owing to its health benefits and pleasant flavor, especially its floral aroma (Ng et al., 2017). Aroma formation in oolong tea mainly results from complicated manufacturing processes (Liu et al., 2018), including solar-withering, turnover, panning, rolling, and drying. To date, about 80 types of volatile aroma compounds have been identified in fresh tea leaves, while more than 300 have been found in oolong tea (Yang et al., 2013). Volatile compounds that constitute oolong aroma are divided into four classes based on their origin, namely, fatty acid derivatives, phenylpropanoids/benzenoids, terpenoids, and norisoprenoids (Fu et al., 2017). Fatty acid (FA) derivatives of carboxylic 6 (C6) volatile compounds are considerably accumulated and transformed during the postharvest processing of oolong tea (Zhou et al., 2020). Postharvest processing imparts the oolong tea with a rich and varied natural floral and fruity aroma. Volatile fatty acid derivatives (VFADs) of C6 compounds, such as hexanal, (*Z*)-3-hexen-1-ol, and hexanoic acid, are not only favorable indicators for tea technicians during oolong tea processing but also precursors of dispersive fruit esters and boiling-point aroma substances.

**Figure 1:**
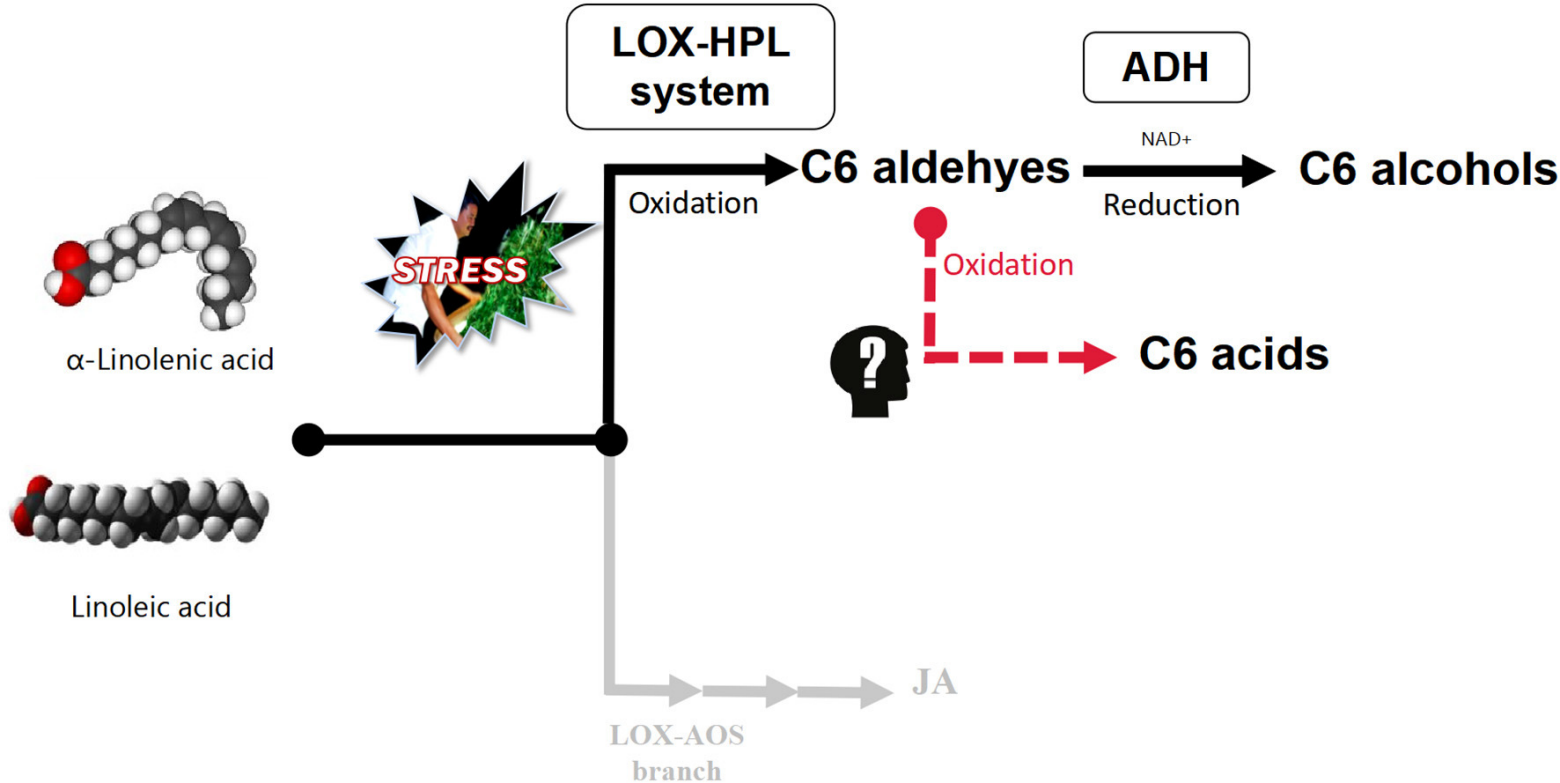
LOX-HPL pathway for C6 compounds derived from long-chain unsaturated fatty acids. Long-chain unsaturated fatty acids (such as α-linolenic acid and linoleic acid) are the precursors of metabolic pathways. The enzyme system composed of LOX (lipoxygenase, EC1.13.11.12) and HPL (hydroperoxide lyase, EC4.1.2.-) oxidatively cleaved long-chain unsaturated fatty acids to form C6 aldehydes, while ADH (alcohol dehydrogenase, EC1.1.1.1) reduced C6 aldehydes to C6 alcohols. However, it was not clear whether C6 aldehydes could form C6 acids through nonenzymatic reactions.

Polyunsaturated fatty acids (PUFAs), α-linolenic acid, and linoleic acid are oxidatively cleaved by the lipoxygenase–hydroperoxide lyase (LOX-HPL) enzyme system under stress to form C6 aldehydes, which have been shown to undergo enzymatic reduction to form C6 alcohols under the control of alcohol dehydrogenase (ADH) aided by NAD+ (Matsui et al., 1991; Qian et al., 2016). However, based on the aroma metabolism profile of pan-fired green tea (Kawakami and Yamanishi, 1999), An'xi Tieguanyin oolong tea (Guo et al., 2021), and flowery black tea (Shi et al., 2018) the metabolic flow of C6 aldehydes relies on the oxygen supply in the upstream of LOX-HPL pathway. Under hypoxic conditions, C6 aldehydes can be reduced to C6 alcohols, which are then esterified, while under normoxic conditions, C6 aldehydes can be oxidized to C6 acids, which are then esterified (Figure 1) (Hao et al., 2016; Qian et al., 2016). Therefore, we speculated that C6 acids, in a competitive relationship with C6 alcohols, accumulate during the postharvest processing of oolong tea.

Presently, there are few reports on the transformation of short-chain aliphatic aldehydes, alcohols, and acids in the plant kingdom. In this study, to verify our prediction and explore the metabolic flow of C6 aldehydes during postharvest processing of oolong tea, normoxic and hypoxic turnover tea leaves were used as research materials with fresh leaves as the control. The dynamics of C6 volatile compounds in manufactured tea leaves, both before and after turnover treatment conducted three times, were compared and analyzed. Furthermore, the response of ADH and its related genes to oxygen in the LOX-HPL pathway was evaluated using an artificial airflow difference. Finally, the transcript levels of hypoxia-induced protein genes were verified. This study aimed to explore the influence of FA metabolism of oolong tea on the formation of aroma quality mediated by airflow factors. The results provided new insights for future research on quality regulation in oolong tea processing.

## Materials and Methods

### Plant Materials and Treatments

**Figure 2:**
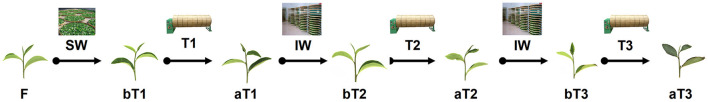
Schematic diagram of sampling points before and after each turnover treatment. F, fresh tea leaves. bT1–bT3, process tea samples before each turnover treatment. aT1–aT3, process tea samples after each turnover treatment. Indoor withering was performed for 30 min between each turnover treatment.

Tea process leaves were collected from *C. sinensis* cv. Huangdan at the educational practicing base of Fujian Agriculture and Forestry University (Fuzhou, China) in July 2018. The plucking standard was “one bud and three leaves,” referring to one bud and three leaves on the same branch, which is usually used throughout oolong tea manufacture in China. The fresh tea leaves (F) were withered under gentle sunlight (26°C, 150,000 lux) during the late evening for 20 min. Afterward, half of the withered leaves (1,000 g) were shaken three times for 5 min, hourly (T1–T3) until fixation. The sampling points were before and after each turnover. The solar-withered leaves (W) were regarded as leaves before the first turnover (bT1), and the tea leaves before the second and third turnover were regarded as bT2 and bT3, respectively. Similarly, the leaves turned over for the first time were regarded as leaves after the first turnover (aT1), and the tea leaves after the second and third turnover were regarded as aT2 and aT3 (Figure 2).

**Figure 3:**
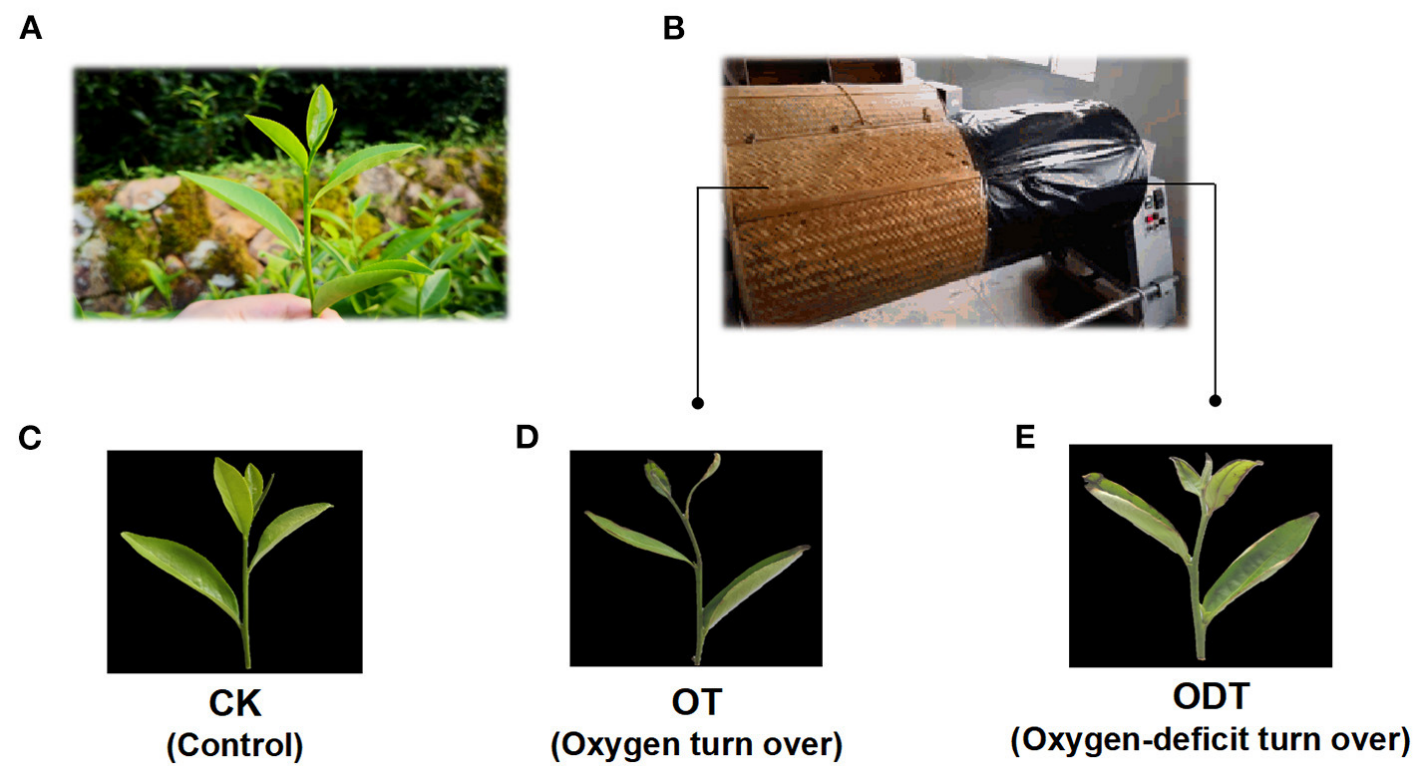
Sampling schematic diagram of oxygen deficiency and normal turnover treatment. **(A)** Fresh leaves were sampled from a tea plant (*Camellia sinensis* cv. Huangdan) in the tea garden. **(B)** *In situ* photographs were taken at the turnover test site. **(C–E)** Apparent imaging of control (CK), oxygen-turnover tea leaves (OT), and oxygen-deficit-turnover tea leaves (ODT).

The remaining withered leaves were subjected to both treatment schemes. The first scheme was an oxygen deficiency treatment. The withered leaves were shaken three times on one side of the turnover machine covered in black plastic film (oxygen-deficit-turnover tea leaves, ODT), while the other side served as a positive control (oxygen-turnover tea leaves, OT), and F acted as a negative control (Figure 3).

Sampling for each treatment above was repeated at least three times. All other samples above were wrapped in tin foil, fixed using the liquid nitrogen sample-fixing method, and stored in an ultra-low temperature refrigerator at −70°C.

### GC-TOF-MS-Based FA-Derived Volatiles

Aroma components were analyzed in accordance with previous studies (Chen et al., 2020), with minor modifications. Processed leaf samples were freeze-dried and ground with a mortar and pestle. The resulting powder (2 g) was transferred to a 20-ml solid-phase microextraction (SPME) bottle and the cap was immediately closed. A 65-μm polydimethylsiloxane/divinylbenzene SPME syringe fiber (Supelco Bel-lefonte, PA, USA) was used for all headspace (HS)-SPME extractions, with an incubation temperature of 80°C, a pre-extraction incubation time of 31 min, and HS-SPME extraction time of 60 min. The aroma components were detected using an Agilent 7890B gas chromatograph (Agilent Co., Santa Clara, CA, USA) coupled with a Pegasus HT time-of-flight mass spectrometer (LECO Co., Saint Joseph, MI, USA) (GC-TOF MS). A capillary column [Rxi-5sil MS, Restek, USA; 30 m × 0.25 mm × 0.25 μm (film thickness)] was employed using high purity helium as the carrier gas at a flow rate of 1 ml/min. Injections were performed in splitless mode using an injection port temperature of 250°C. The transfer line temperature was set to 275°C. The GC oven temperature program was initially held at 50°C for 5 min, followed by a temperature ramp of 3°C/min to 210°C with a 3-min hold, and a final temperature ramp of 15°C/min to 230°C. The MS operating parameters were as follows: ionization potential, −70 eV; ion source temperature, 250°C; acquisition voltage, 1,530 V; mass range, 30–500 amu; and 10 spectra/s.

### ADH Activity Analysis

Alcohol dehydrogenase activity was monitored using an ultraviolet-visible spectrophotometer (Perkin-Elmer, Waltham, MA, USA). Enzyme activity measurements were performed using an ADH activity detection kit following the instructions of the manufacturer (Beijing Solarbio Science & Technology Co., Ltd., China). The ADH activity was calculated using the mass of the original sample, as reported previously (Zhou et al., 2020).

### Source of Omics Data

The target gene sequence and functional annotation were based on reference tea plant genome sequences downloaded from the Tea Plant Information Archive (TPIA; http://tpia.teaplant.org/) (Xia et al., 2020). Fragments per kilobase of transcript per million mapped reads (FPKM) values and primer design were based on transcriptome sequencing data previously generated in our laboratory (pending submission). The protein annotation sequence (CSSChrLev20200506PEP) of the chromosome-level genome of *C. sinensis* cv. Shuchazao was downloaded from TPIA, the Stockholm file of the Hidden Markov Model (HMM) of the HIG_1_N domain was obtained from the Pfam database (http://pfam.xfam.org/), and then a seed file of PF04588 was generated. The “hmmbuild” program in the hmmer-3.0-window software package (Eddy and Pearson, 2011) (http://eddylab.org/software/hmmer3/3.0/) was used to build and complete the establishment of the HMM file of HIG. The “hmmsearch” program in the same software package was run to obtain eight candidate *CsHIG* genes, which were denoted *CsHIG1*–*CsHIG8*.

### Gene Expression Analysis

Lyophilized process samples (0.1 g) were ground to powder in liquid nitrogen. Total RNA was extracted from the frozen powder using the RNAprep Pure Plant Kit (Tiangen Biotech Co. Ltd., Beijing, China). cDNA was generated with the PrimeScript RT Reagent Kit with a gDNA Eraser (TaKaRa Biotech Co., Ltd., Dalian, China). Quantitative real-time PCR (qRT-PCR) was performed as previously described using a LightCycler 480 Real-Time PCR System (Roche Molecular Systems, Alameda, CA, USA) (Zhou et al., 2020). The reaction mixture had a total volume of 20 μL, comprising cDNA (1 μL), 2 × TB Green Premix Ex Taq II (TaKaRa; 10 μL), 10 μM forward primer (0.8 μL), 10 μM reverse primer (0.8 μL), and double-distilled water (ddH_2_O, 7.4 μL). Amplification reactions were conducted as follows: denaturation for 10 s at 95°C, 40 cycles of 5 s at 95°C, and 20 s between 55 and 60°C using the Tm function of the primers. Fluorescent detection was performed after each extension step. Amplification reactions were conducted as follows: denaturation for 10 s at 95°C, 40 cycles of 5 s at 95°C, and 20 s between 55 and 60°C using the Tm function of the primers. Fluorescent detection was performed after each extension step. The relative expression values were calculated using the comparative CT (2^−ΔΔCT^) method (Livak and Schmittgen, 2001). *CsGAPDH* (NCBI accession number: JKA295375.1) was used as a reference gene. Primer sequences of selected genes were designed by DNAMAN version 7 (Lynnon Biosoft, Vaudreuil, Canada) and are listed in Supplementary Table 1, including CSA019598, *CsADH*, CL822.Contig4, CsHIF4, and CsHIF6. Primer synthesis was performed by TSINGKE Co., Ltd (Beijing, China).

### Statistical Analysis

All experimental results are provided as mean ± standard error of the mean (SEM). Statistical analysis was conducted using SPSS (PASW Statistics Base 18, IBM, Chicago, IL, USA) to determine significance. Statistical differences between measurements were assessed using Tukey's test (^*^P < 0.05, ^**^P < 0.01) after analysis of variance. All figures were generated using Prism (GraphPad, Version 6.01, GraphPad Software Inc, San Diego, CA, USA).

## Results and Discussion

### Changes in C6 Volatile Compounds in LOX-HPL Pathway During Turnover and Indoor-Withering Process

**Figure 4:**
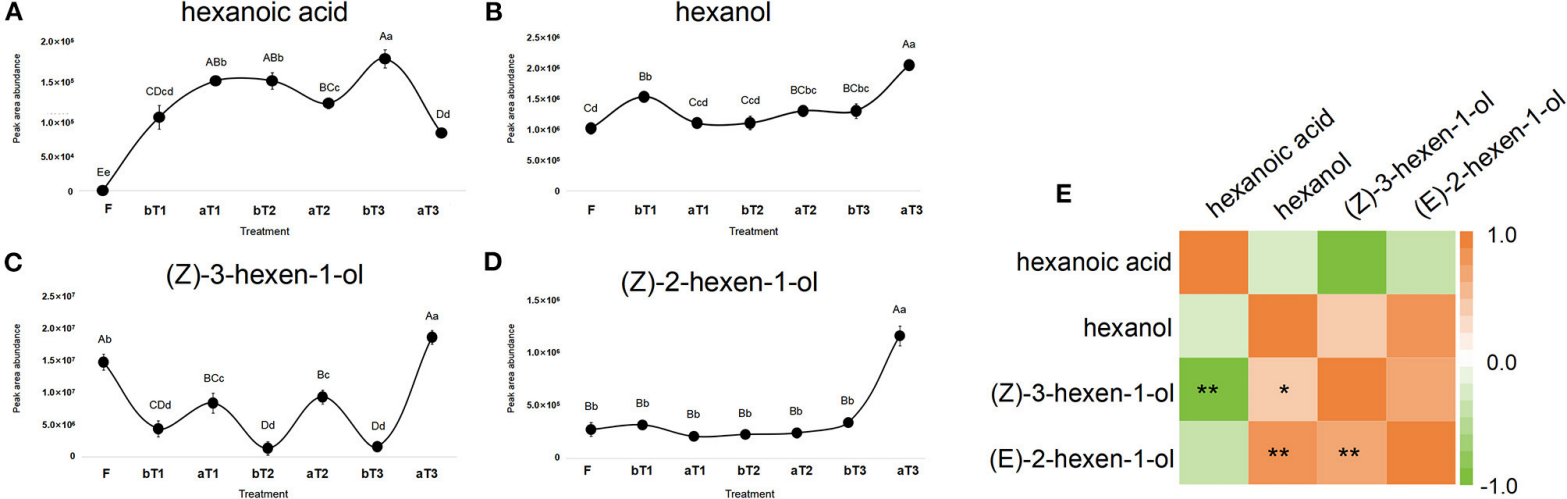
Dynamic changes in C6 acid and C6 alcohols peak area abundance and correlation heat map during postharvest processing of oolong tea. **(A–D)** Dynamic changes of **(A)** hexanoic acid, **(B)** hexanol, **(C)** (*Z*)-3-hexen-1-ol, and **(D)** (*Z*)-2-hexen-1-ol. **(E)** Analysis of correlation heat map between C6 acid and C6 alcohols. F, fresh tea leaves; bT1–bT3, tea leaves before the three turnover treatments; aT1–aT3, tea leaves after the three turnover treatments. Different capital letters (A–D) and lowercase letters (a–d) represent significant differences at the *p* < 0.01 and *p* < 0.05 levels using Tukey's test, respectively. One or two asterisks denote a statistically significant difference (*P < 0.05; **P < 0.01) in relative amount between C6 acid and C6 alcohols during the turnover process of oolong tea.

Turnover plays a critical role in the formation of the natural floral and fruity aroma of oolong tea (Zeng et al., 2016). A large number of C6 volatile compounds represented by GLVs (green leaf volatiles) are formed and transformed during the turnover process (Ono et al., 2016). GLVs can be transformed into terpenoid volatiles and provide signaling substances generated by abiotic stress (Hu et al., 2018). Targeted detection of C6 acids and C6 alcohols during the postharvest processing of oolong tea showed that three types of C6 alcohol were detected, namely, hexanol (CAS: 111-27-3), (*Z*)-3-hexen-1-ol (CAS: 928-96-1), and (*E*)-2-hexen-1-ol (CAS: 928-95-0), with hexanoic acid (CAS: 142-62-1) as the only C6 acid detected. Both the C6 acid and C6 alcohols showed an overall increasing trend during the postharvest processing of oolong tea. Among them, the C6 alcohol contents had increased significantly after the third turnover, while hexanoic acid showed the opposite trend. Notably, the turnover stage is conducive to increasing the C6 alcohol content, while the spreading stage promotes C6 acid accumulation. Peak areas representing the total abundance of (*Z*)-3-hexen-1-ol and hexanoic acid showed an increasing trend during the postharvest processing of oolong tea (Figures 4A–D), benefiting from sufficient C6 aldehydes from the oxidation and cleavage of PUFAs by the LOX-HPL enzyme system. Correlations were statistically assessed using Spearman's test. A highly significant negative correlation was noted between the peak areas representing the abundances of (*Z*)-3-hexen-1-ol and hexanoic acid (*r* = −0.881, *p* < 0.01) (Figure 4E). Therefore, a competitive relationship might exist between C6 alcohols and C6 acids during the postharvest process of oolong tea. In previous studies, C_6_ volatile compounds and their derivatives exhibited disparate changing trends under turnover and indoor-withering treatment (Chen et al., 2018). Furthermore, the activity of LOX, a rate-limiting enzyme in the LOX pathway localized in the chloroplast, can respond to turnover (Liu et al., 2018; Chen et al., 2021). Our research group has reported that C6 volatiles and the structural genes of the LOX pathway can respond to mechanical force during a turnover to form aliphatic aromas in oolong tea (Zhou et al., 2019a,b). However, the nature of the difference between (*Z*)-3-hexen-1-ol and hexanoic acid is relatively unusual and needs to be further explored.

### Analysis of ADH and *CsADH* Gene Expression Patterns Under Hypoxic Turnover Conditions

**Figure 5:**
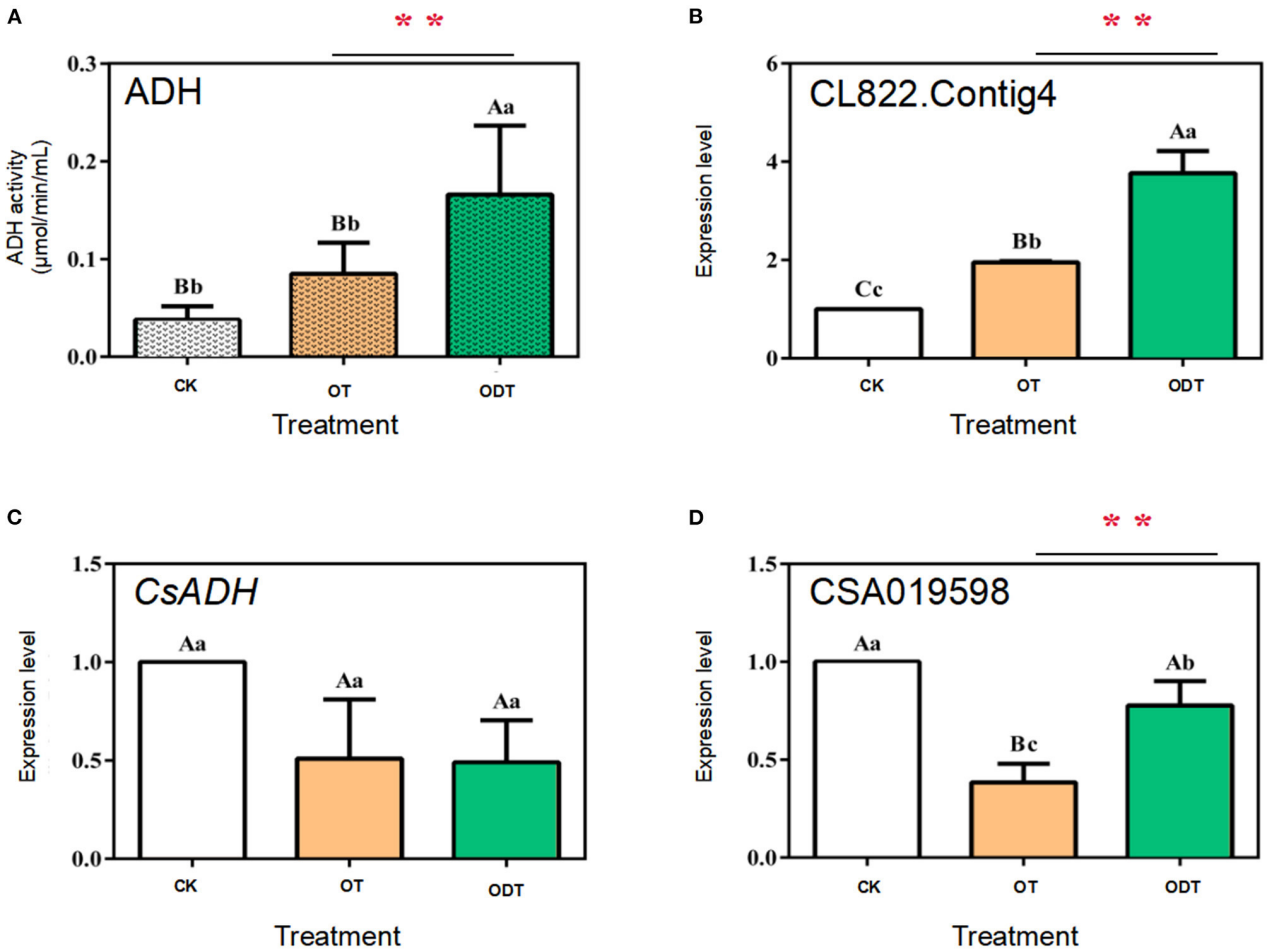
ADH enzyme activity and relative expression levels of three *CsADH* genes under normoxic and hypoxic conditions during the turnover process of oolong tea. **(A)** Enzyme activity of ADH (EC 1.1.1.1); **(B–D)** expression levels of **(B)** CSA019598, **(C)** CSA019598 of *CsADH* (GenBank accession no. HM440157.1), and **(D)** CL822.Contig4. Data are expressed as means ± standard errors of the mean of six independent experiments. Different uppercase letters (*P* < 0.01) and lowercase letters (*P* < 0.05) indicate significant differences among different treatments from Tukey's test. CK (control group) represents fresh tea leaves without any treatment. ODT (oxygen-deficient leaves) represents fresh tea leaves turned over under hypoxic conditions. OT (normal leaves) represents fresh tea leaves turned over under normoxic conditions. Two asterisks denote a statistically significant difference (**P < 0.01) in relative amount between ODT and OT during the postharvest processing of oolong tea.

ADH (alcohol dehydrogenase, EC 1.1.1.1), a zinc-containing enzyme involved in short-chain alcohol metabolism in the biological kingdom, reduces C6 aldehydes to C6 alcohols under biotic and abiotic stress in the LOX-HPL pathway (Cirilli et al., 2012). However, C6 aldehydes might be further oxidized to form C6 acids under normoxic conditions. Therefore, the reduction of C6 aldehydes to C6 alcohols by ADH seems to require hypoxic conditions. Changes in external airflow factors can create normoxic or hypoxic conditions during turnover, which might mediate *CsADH* gene transcription levels and ADH activity, affecting the formation of, and changes in, fatty acid aroma components during postharvest processing of oolong tea. The ADH activity and expression levels of three *CsADH* genes in CK, OT, and ODT were measured. The ADH activity was the highest in ODT (0.16 μmol/min/mL), followed by OT (0.09 μmol/min/mL), and lowest in CK (0.03 μmol/min/mL). The ADH activity in ODT was significantly higher than those in CK and OT (*P* < 0.01) (Figure 5A). CK, ODT, and OT showed transcription levels of the *CsADH* genes. Three ADH regulatory genes were selected based on previous studies, designated as CSA019598, *CsADH*, and CL822.Contig4 (Zhou et al., 2020). The CL822.Contig4 expression level of ODT was significantly higher than those of CK (*P* <0.01) and OT (*P* <0.05), which was significantly positively correlated with the change in ADH activity (Figure 5B). However, a different expression pattern was observed for CSA019598 and *CsADH*. These two genes showed the highest expression levels in CK. In OT and ODT, the *CsADH* gene expression level decreased to similar levels (Figures 5C,D). However, the CSA019598 expression level decreased significantly and extremely significantly in ODT (0.773) and OT (0.383), respectively. As indicated above, turnover under hypoxic conditions might stimulate ADH activity. CL822.Contig4 might be a key regulatory gene during the postharvest processing of oolong tea. In a previous study, *CsADH* gene expression levels firstly increased and then decreased as the manufacturing process progressed (Hu et al., 2018). However, the potential candidate gene (CSA019598), previously acquired by our research group (Zhou et al., 2020), showed no significant correlation with changes in ADH activity. This might be attributed to the process mode, processing room environment, or even the tea growing season (Sekiya et al., 1984). Based on the comparison of the tea tree genome (CSS, CSA), we found that the gene did not belong to the ADH family, but the 2-alkenal reductase (AER) family. Both AER and ADH genes belonged to the alcohol dehydrogenase (MDR) superfamily. These two amino acid sequences have high similarities (Persson et al., 2008). CsADH gene was one of the members of the AER gene family (Xia et al., 2020). This may be the main reason for the opposite trend of this gene and CSA019598.

### Analysis of FA-Derived Volatiles in Oolong tea Under Hypoxic Turnover Conditions

**Figure 6:**
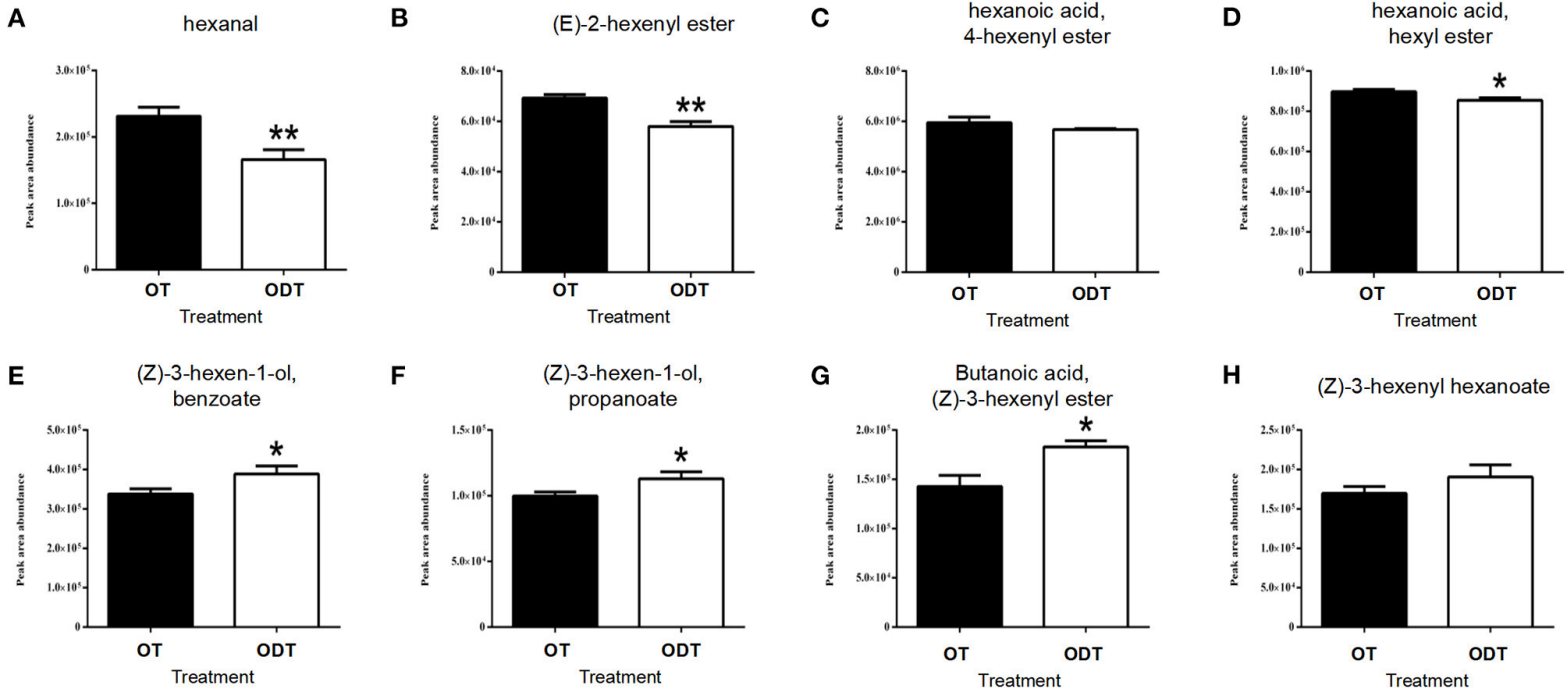
Contents of volatile fatty acid derivatives in raw tea under normoxic and hypoxic conditions during the turnover process of oolong tea. **(A)** Hexanal, **(B)** (*E*)-2-hexenyl ester, **(C)** 4-hexenyl hexanoate, **(D)** hexyl hexanoate, **(E)** (*Z*)-3-hexen-1-yl benzoate, **(F)** (*Z*)-3-hexen-1-yl propanoate, **(G)** (*Z*)-3-hexenyl butanoate, and **(H)** (*Z*)-3-hexenyl hexanoate. Asterisks denote statistically significant differences (*p < 0.05; **p < 0.01) between relative amounts of OT and ODT. Error bars indicate the standard error (SE) of the mean.

VFAD-targeted analysis of oolong tea manufactured under normoxic and hypoxic conditions (Figure 6) showed that hexanal was detected among C6 aldehydes, with the content (2.31 × 10^5^) in OD being significantly higher than that in ODT (1.66 × 10^5^). Hexanal is the oxidative product of PUFAs in the LOX-HPL enzyme system. This system is beneficial to the oxidative cleavage of substrates under normoxic conditions, thus promoting hexanal accumulation. Hexanoic acid and hexanol were not detected in the manufactured oolong tea and might have been transformed or lost during the fixation, rolling, and drying processes. However, esters derived from hexanoic acid and hexanol indirectly reflected the metabolic flow of hexanal in ODT and OT. Hexanoic acid esters (HAEs) detected were (*E*)-2-hexenyl hexanoate (CAS: 53398-86-0), 4-hexenyl hexanoate (CAS: 31501-11-8), and hexyl hexanoate (CAS: 123-66-0). The contents of these three esters in OT were higher than those in ODT. Among them, the contents of (*E*)-2-hexenyl hexanoate, (7.03 × 10^4^) and hexyl hexanoate (9.1 × 10^5^) in OT were extremely significantly (*P* < 0.01) and significantly (*P* < 0.05) higher than those in ODT (5.59 × 10^4^ and 8.41 × 10^5^, respectively). In contrast, hexanol-derived esters (HAEs) measured included (*Z*)-3-hexen-1-yl benzoate (CAS: 25152-85-6), (*Z*)-3-hexen-1-yl propanoate (CAS: 33467-74-2), (*Z*)-3-hexenyl butanoate (CAS: 16491-36-4), and (*Z*)-3-hexenyl hexanoate (CAS: 31501-11-8). Notably, the contents of these four compounds in OT were lower than those in ODT. Except for (*Z*)-3-hexenyl hexanoate, compounds (*Z*)-3-hexen-1-yl benzoate (3.38 × 10^5^), (*Z*)-3-hexen-1-yl propanoate (9.63 × 10^4^), and (*Z*)-3-hexenyl butanoate (1.43 × 10^5^) had significantly (*P* < 0.05) lower contents in OT than in ODT (3.85 × 10^5^, 1.15 × 10^4^, and 1.86 × 10^5^, respectively). Therefore, OT treatment was conducive to the accumulation of HAEs, while ODT treatment was conducive to the accumulation of HEs.

### Screening and Analysis of *CsHIP* Genes and Their Expression Patterns Under Normoxic and Hypoxic Conditions During Turnover

Hypoxia-induced protein is a common signal protein responding to hypoxic stress in higher eukaryotes. Current research on the stable expression of hypoxia-induced factor (HIF)-related genes depends on oxygen deficiency in mammals. HIF-α, a subclass of HIF, is hydroxylated with sufficient oxygen and ubiquitinated by von Hippel–Lindau disease and degradation (Kaelin, 2017). HIF-α is not degraded with increasing oxygen concentration in the atmosphere, promoting the expression of a hypoxia target gene and enhancing cell adaptability (Gregg et al., 2013). To further demonstrate the response degree of manufactured tea leaves to oxygen factors in normoxic and hypoxic microenvironments, whole-genome identification was conducted for the *CsHIG* gene family, and the potential key transcripts of *CsHIG* genes were discovered.

Information on the candidate *CsHIG* gene and its main physical and chemical properties are shown in Supplementary Table 2. The results showed that 8 *CsHIG* gene family members were scattered on the chromosomes. The length of the gene coding sequence (CDS) was 297–303 bp, except for the CsHIG7 member. The number of amino acids in the encoded protein was 98–100 and the molecular weight range was 11,013.71–11,084.74 Da, with CsHIG3 protein having the largest number of amino acids and highest molecular weight. The isoelectric point (pI) distribution range of the protein sequences encoded by the eight *CsHIG* gene family members was 9.07–10.21, and the pI value of CsHIF5 was the largest (10.21). The aliphatic index ranged from 86.00 to 100.71.

**Figure 7:**
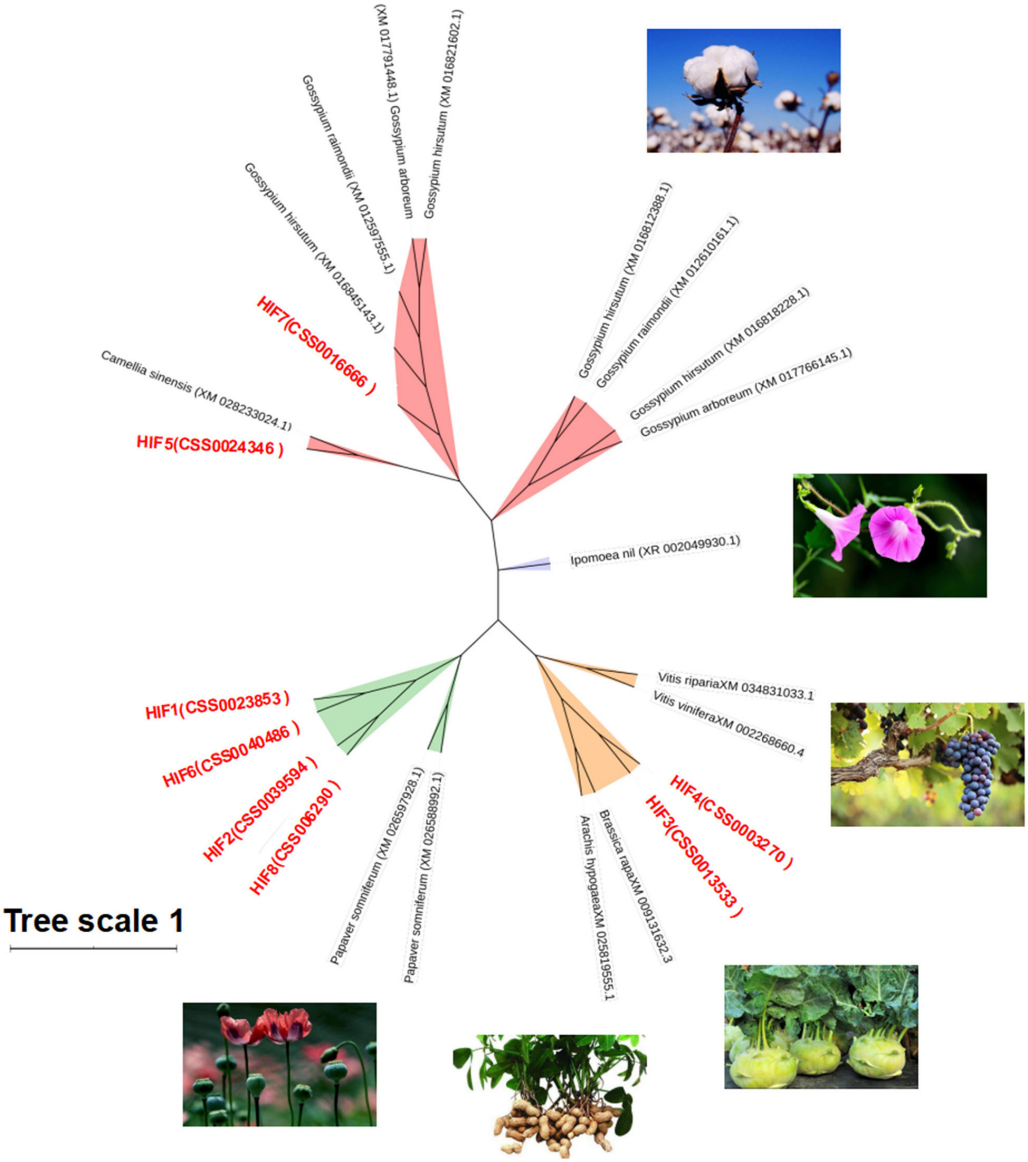
Phylogenetic tree based on amino acid sequence homology of CsHIG proteins. Phylogenetic tree of 8 *HIP* genes in the tea plant and 16 ADH genes (green) in six other species: Poppy (*Papaver somniferum*, XM_026597928.1 and XM_026588992.1); peanut (*Arachis hypogaea*, XM_025819555.1); turnip (*Brassica rapa*, XM_009131632.3); grape (*Vitis vinifera*, XM_002268660.4; *Vitis riparia*, XM_034831033.1); Morning glory (Ipomoea nil, XR 002049930.1); and cotton (*Gossypium arboreum*, XM_017766145.1 and XM_017791448.1; *Gossypium hirsutum*, XM_016818228.1, XM_016845143.1, XM_016812388.1, and XM_016818228.1; and *Gossypium raimondii*, XM_012610161.1, XM_012597555.1, and XM_012610161.1).

To understand the genetic relationship and evolutionary distance between *CsHIG* gene family members and *HIG* genes of other species, BLASTn homology comparison analysis was performed on the amino acid sequences of the eight tea HIG gene-encoded proteins obtained from the National Center for Biotechnology Information (NCBI) screening. The HIG protein amino acid sequences of cotton, poppy, turnip, peanut, and other species were obtained, and a phylogenetic tree was constructed, as shown in Figure 7. BLASTn analysis indicated that *CsHIG5* was clustered with HIG1 (D7SHV8.1) and HIG1 (D7TWS7.1) from grape (*Vitis vinifera*); *CsHIG7* showed high homology with HIG1 from grape (*Vitis vinifera*) and cotton (*Gossypium hirsutum, Gossypium raimondii*, and *Gossypium arboreum*). Furthermore, CsHIG2, CsHIG3, CsHIG4, and CsHIG8 showed high sequence homology among the members and were genetically similar to peanuts (*Arachis hypogaea*) and grapes (*Vitis vinifera, Vitis riparia*), while CsHIG1 and CsHIG6 showed high homology with HIG from cotton (*Gossypium hirsutum, Gossypium raimondii*, and *Gossypium arboreum*), poppy (*Papaver somniferum*), and turnip (*Brassica rapa*).

**Figure 8:**
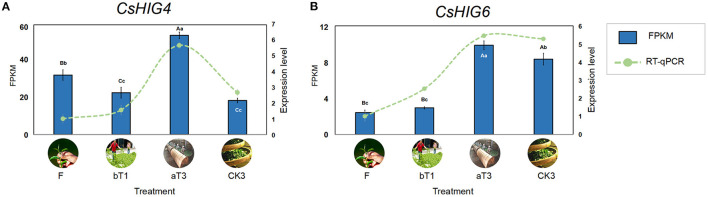
FPKM values and RT-qPCR test verification of key *CsHIG* gene transcripts during postharvest processing of oolong tea. FPKM values and expression levels of **(A)** *CsHIG4* and **(B)** *CsHIG6*. F, Fresh tea leaves; bT1, tea leaves before first turnover treatment; aT3, tea leaves after third turnover treatment; CK3, control of aT3 without turnover treatment. Different uppercase letters (*P* < 0.01) and lowercase letters (*P* < 0.05) indicate significant differences among different treatments from Tukey's test. Error bars indicate the standard error (SE) of the mean.

Based on the transcript coding region sequence cds.fa database during the postharvest processing of oolong tea, the results of local BLAST of eight *CsHIG* gene family members indicated that the *CsHIG1* and *CsHIG6* scores compared with the transcript Unigene1790_All were 571 and 595, with the similarity reaching 99% and 100%, respectively. Meanwhile, the comparison scores of *CsHIG1* and *CsHIG6* with the transcript CL10755.Contig2_All were 577 and 585, respectively, with similarities for both of 99%. According to the principle that a greater matrix score results in a smaller expected value (E_value), greater identities, and a greater degree of matching, CL10755.Contig2_All and Unigene1790_All were screened as key transcripts of *CsHIG6* and *CsHIG4* genes, respectively (Supplementary Table 3). The RNA-Seq results showed that peak expression levels of the two key transcripts all appeared in turnover leaves (T3). The FPKM of CL10755.Contig2_All in T3 was 1.66, 2.37, and 2.91 times those of the fresh leaves (F), withered leaves (W), and non-turnover leaves (CK3), respectively, with an extremely significant difference between each other (*P* < 0.01). Similarly, the FPKM of Unigene1790_All in T3 was 4.07, 3.45, and 1.18 times those of F, W, and CK3, respectively, with an extremely significant difference between each other (*P* < 0.01). The results of FPKM verification using RT-qPCR technology showed that the relative expression levels of CL10755.Contig2_All and Unigene1790_All tended to be consistent with FPKM during the postharvest processing of oolong tea (*r* = 0.743^**^, *r* = 0.959^**^. ^**^Indicates that there was an extremely significant correlation between FPKM and relative expression level) (Figure 8). Therefore, the key transcripts screened, CL10755.Contig2_All and Unigene1790_All of the *CsHIG* gene showed a hypoxic stress response during the turnover process of oolong tea.

**Figure 9:**
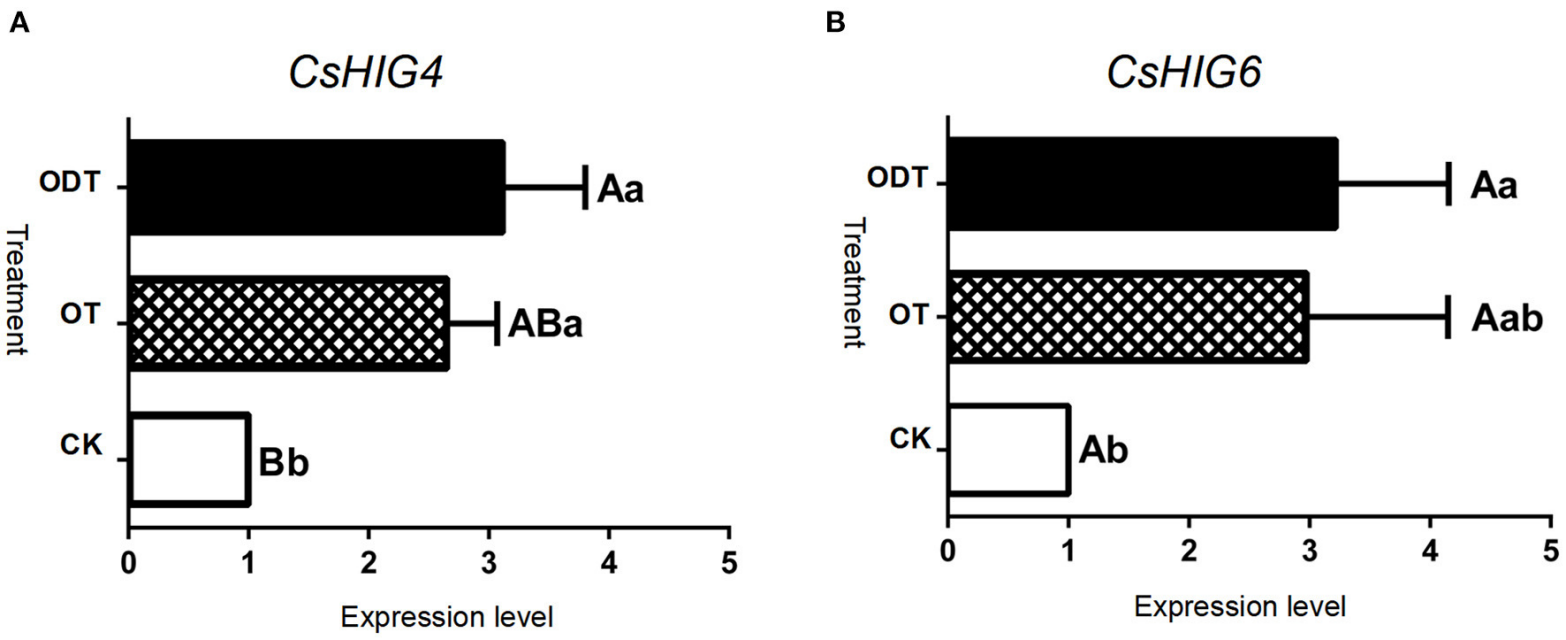
Relative expression level of key *CsHIG* gene transcripts under normoxic and hypoxic conditions during the turnover process of oolong tea. FPKM values and expression levels of **(A)** *CsHIG4* and **(B)** *CsHIG6*. F (control group) represents fresh tea leaves without any treatment. ODT (oxygen-deficient leaves) represents fresh tea leaves turned over under hypoxic conditions. OT (normal leaves) represents fresh tea leaves turned over under normoxic conditions. Different uppercase letters (*P* < 0.01) and lowercase letters (*P* < 0.05) indicate significant differences among different treatments from Tukey's test. Error bars indicate the standard error (SE) of the mean.

To further demonstrate the influence of the turnover microenvironment on *CsHIG* gene expression, fresh tea leaves (CK), hypoxic-turnover leaves (ODT), and normoxic-turnover tea leaves (OT) were used as materials. The results showed that, compared with CK, key transcript CL10755.Contig2_All of the *CsHIG4* gene was increased 2.65- and 3.12-fold in ODT and OT, respectively. The CL10755.Contig2_All expression levels of ODT and OT were extremely significantly (*P* < 0.01) and significantly higher (*P* < 0.05) than those of CK, respectively, but no significant difference was observed between ODT and OT (*P* > 0.05). Similar to CL10755.Contig2_All, key transcript Unigene1790_All of the *CsHIG6* gene was increased 2.98- and 3.22-fold in ODT and OT compared with CK, respectively. The Unigene1790_All expression level in ODT was significantly higher than that in fresh leaves (*P* > *0.0*5) (Figure 9). Therefore, we have further clarified the hypoxic microenvironment created by turnover treatment, and we have screened and obtained key transcripts of *CsHIG4* and *CsHIG6* genes that responded to turnover treatment during the postharvest processing of oolong tea.

## Conclusion

Based on the conversion law of the compound, we concluded that the C6 aldehydes of the LOX-HPL pathway might undergo oxidation reactions to form C6 acids and their derivative esters, such as hexanoic acid, (E)-2-hexenyl ester, hexanoic acid, 4-hexenyl ester, and hexanoic acid in a micro-environment with abundant oxygen supply through non-enzymatic reactions during the postharvest process of oolong tea; while, when the micro-environmental oxygen was insufficient, hypoxia-induced protein (HIP) genes would be transcribed, and ADH enzyme activity and related gene expression were up-regulated, promoting the conversion of C6 aldehydes to C6 alcohols and their derivative esters, such as (Z)-3-hexen-1-ol, benzoate, (Z)-3-hexen-1-ol, propanoate, Butanoic acid, (Z)-3-hexenyl ester, and (Z)-3-hexenyl hexanoate through enzymatic reduction reactions through non-enzymatic reactions during the postharvest process of oolong tea. In summary, the difference in oxygen factors in the micro-environment might mediate the metabolic flow of C6 aldehydes in the LOX-HPL pathway and then affect the formation and proportion of fatty acid esters of oolong tea.

## Data Availability Statement

The original contributions presented in the study are included in the article/Supplementary Materials, further inquiries can be directed to the corresponding author/s.

## Author Contributions

Z-wZ and YS conceived and designed the experiments. Z-wZ, Q-yW, Q-cH, YY, and H-lD performed the experiments. Z-xN, Z-xL, YS, and N-xY analyzed the data. Z-wZ, W-jB, Q-yW, and H-lD interpreted the results. Z-wZ, Q-yW, and Z-xL wrote the manuscript. All authors discussed the results and reviewed the final manuscript.

## Funding

This research was supported by China Agriculture Research System of MOF and MARA (CARS-19), and the Fujian Agriculture and Forestry University Science and Technology Innovation Fund (CXZX2017178). The funders played no role in the study design, data collection, and analysis, the decision to publish, or manuscript preparation.

## Conflict of Interest

The authors declare that the research was conducted in the absence of any commercial or financial relationships that could be construed as a potential conflict of interest.

## Publisher's Note

All claims expressed in this article are solely those of the authors and do not necessarily represent those of their affiliated organizations, or those of the publisher, the editors and the reviewers. Any product that may be evaluated in this article, or claim that may be made by its manufacturer, is not guaranteed or endorsed by the publisher.
