# Supplementary material for: Metabolic Flow of C6 Volatile Compounds From LOX-HPL Pathway Based on Airflow During the Post-harvest Process of Oolong Tea
Source: Front Plant Sci. 2021 Oct 22;12:738445. doi: 10.3389/fpls.2021.738445 (PMC8569582; doi:10.3389/fpls.2021.738445)
Supplement: Supplementary file 1 [file Table_1.DOC]

**TABLE S1. The specific primers of mRNA for RT-qPCR analysis**

| Gene name | Accession | Base sequence (5’-3’) | Amplification length（bp） | Efficiency value (%) |
| --- | --- | --- | --- | --- |
| *CsADH* | HM440157.1 | F: TGTTGGAAGTGCTGGAACC | 220 | 98.5 |
|  |  | R: CATACCACACACGGCAATG |  |  |
| CSA019598 | CSA019598 | F: CCCTTTGCCACACTGATGT | 107 | 99.0 |
|  |  | R: CTCGCCTACACTTTCCACAAT |  |  |
| *CL822.Contig4* | CL822.Contig4 | F: GCCATCTTTGATTGGAATGG | 175 | 100.4 |
|  |  | R: GGTGCCACAACCTTGATCTT |  |  |
|  |  | R: CAGTGGGAACACGGAAAGC |  |  |
| *CsHIF4* | CL10755.Contig2 | F: ATGGCTGAGCGGTATTACG | 161 | 99.8 |
|  |  | R: CTCCCGATTTGTGGTCATAG |  |  |
| *CsHIF6* | Unigene1790 | F: GCTGAAGCGAAGAACACCA | 113 | 100.8 |
|  |  | R: TTGTAGGCGATTGAACCCG |  |  |
| *CsGADPH* | [KA295375.1](http://www.ncbi.nlm.nih.gov/nuccore/KA295375.1) | F: TTGGCATCGTTGAGGGTCT | 206 | 100.3 |
|  |  | R: CAGTGGGAACACGGAAAGC |  |  |
