# Supplementary material for: Metabolic Flow of C6 Volatile Compounds From LOX-HPL Pathway Based on Airflow During the Post-harvest Process of Oolong Tea
Source: Front Plant Sci. 2021 Oct 22;12:738445. doi: 10.3389/fpls.2021.738445 (PMC8569582; doi:10.3389/fpls.2021.738445)
Supplement: Supplementary file 2 [file Table_2.DOC]

**TABLE S2 The information and physical and chemical property of eight selected *CsHIG* gene family members**

| Name | CSS ID | Location | [initiation](../../../../D:/360Downloads/Youdao/Dict/8.9.6.0/resultui/html/index.html#/javascript:;) [site](../../../../D:/360Downloads/Youdao/Dict/8.9.6.0/resultui/html/index.html#/javascript:;) | termination site | CDS length  (bp) | amino acids（a.a.） | molecular weight（Da） | pI | aliphatic index |
| --- | --- | --- | --- | --- | --- | --- | --- | --- | --- |
| *CsHIG1* | CSS0023853 | Contig1125 | 32748 | 34821 | 300 | 99 | 11104.69 | 9.46 | 89.80 |
| *CsHIG2* | CSS0039594 | Chr9 | 7923425 | 7926117 | 297 | 98 | 11013.71 | 9.51 | 100.71 |
| *CsHIG3* | CSS0013533 | Contig979 | 51609 | 53658 | 303 | 100 | 11084.74 | 9.48 | 89.90 |
| *CsHIG4* | CSS0003270 | Chr3 | 62180457 | 62182730 | 303 | 100 | 11164.78 | 9.07 | 86.00 |
| *CsHIG5* | CSS0024346 | Chr2 | 7604494 | 7605136 | 300 | 99 | 11185.08 | 10.21 | 89.60 |
| *CsHIG6* | CSS0040486 | Chr5 | 186859304 | 186861582 | 300 | 99 | 11077.66 | 9.46 | 89.80 |
| *CsHIG7* | CSS0016666 | Chr1 | 47262412 | 47263731 | 234 | 77 | 8449.78 | 9.70 | 96.49 |
| *CsHIG8* | CSS0006290 | Chr9 | 8190895 | 8193305 | 297 | 98 | 11013.71 | 9.51 | 100.71 |
