# Supplementary material for: Metabolic Flow of C6 Volatile Compounds From LOX-HPL Pathway Based on Airflow During the Post-harvest Process of Oolong Tea
Source: Front Plant Sci. 2021 Oct 22;12:738445. doi: 10.3389/fpls.2021.738445 (PMC8569582; doi:10.3389/fpls.2021.738445)
Supplement: Supplementary file 3 [file Table_3.DOC]

**Table S3 Result of local blast of eight HIG gene based on transcriptome database**

| Gene name | CSS ID | Transcript | Score (bits) | E_value | Identities | Annotation (pfam_desc) |
| --- | --- | --- | --- | --- | --- | --- |
| *CsHIG1* | CSS0023853 | Unigene1790_All | 571 (288) | e^-162^ | 297/300 (99%) | Hypoxia induced protein conserved region |
| *CsHIG2* | CSS0039594 | None | | | | |
| *CsHIG3* | CSS0013533 | CL10755.Contig2_All | 577 (291) | e^-164^ | 300/303 (99%) | Hypoxia induced protein conserved region |
| *CsHIG4* | CSS0003270 | CL10755.Contig2_All | 585 (295) | e^-166^ | 301/303 (99%) |
| *CsHIG5* | CSS0024346 | None | | | | |
| *CsHIG6* | CSS0040486 | Unigene1790_All | 595 (300) | e^-169^ | 300/300 (100%) | Hypoxia induced protein conserved region |
| *CsHIG7* | CSS0016666 | None | | | | |
| *CsHIG8* | CSS0006290 | None | | | | |
